# The interaction of QRS duration with cardiac magnetic resonance derived scar and mechanical dyssynchrony in systolic heart failure: Implications for cardiac resynchronization therapy^☆^

**DOI:** 10.1016/j.ijcha.2017.11.005

**Published:** 2017-12-13

**Authors:** Tom Jackson, Sana Amraoui, Manav Sohal, Eva Sammut, Jonathan M. Behar, Simon Claridge, Jessica Webb, Ben Sienecwicz, Reza Razavi, Christopher Aldo Rinaldi, Gerald Carr-White

**Affiliations:** a King's College London, London, United Kingdom; b Hopital Haut-Lévêque, Pessac, France; c Guy's and St. Thomas' Hospitals, London, United Kingdom

**Keywords:** Narrow QRS, Cardiac resynchronization therapy, Dyssynchrony, Cardiac magnetic resonance imaging

## Abstract

**Background:**

Trials using echocardiographic mechanical dyssynchrony (MD) parameters in narrow QRS patients have shown a negative response to CRT. We hypothesized MD in these patients may relate to myocardial scar rather than electrical dyssynchrony.

**Methods:**

We determined the prevalence of cardiac magnetic resonance (CMR) derived measures of MD in 130 systolic heart failure patients with both broad (≥ 130 ms - BQRS) and narrow QRS duration (< 130 ms - NQRS). We assessed whether late gadolinium enhancement derived scar might explain the presence of MD amongst narrow QRS patients. Dyssynchrony was calculated on the basis of a systolic dyssynchrony index (SDI).

**Results:**

Fifty-nine patients (45%) had a NQRS and the remaining had QRS ≥ 130 ms (BQRS group). 25% of NQRS patients had MD based on SDI. In all narrow and broad QRS patients with MD there was a significantly lower scar volume than those without MD (7.4 ± 10.5% vs 13.7 ± 13.3% vs. p < 0.01). This was the case in the BQRS group with a significantly lower scar burden in patients with MD (5.0 ± 7.7% vs 15.4 ± 15.6%, p < 0.01). Notably in the NQRS group this difference was absent with an equal scar burden in patients with MD 13.3 ± 13.9% and without MD 12.5 ± 11%, p = 0.92.

**Conclusions:**

25% of patients with systolic heart failure and a NQRS (< 130 ms) have CMR derived mechanical dyssynchrony. Our findings suggest MD in this group may be secondary to myocardial scar rather than electrical dyssynchrony and therefore not amenable to correction by CRT. This may give insight into non-response and potential harm from CRT in this group.

## 1 Introduction

Cardiac resynchronization therapy (CRT) is a an effective treatment for patients with systolic heart failure and a broad QRS duration, however most heart failure patients have narrow QRS [1]. If CRT could be applied successfully to a proportion of these patients the population effects would be very large. Imaging derived mechanical dyssynchrony (MD) parameters have been excluded from recent CRT guidelines [2] due to their poor performance and reproducibility in the PROSPECT study [3]. The assessment of mechanical dyssynchrony amongst patients with narrow QRS has demonstrated its significant prevalence [4], [5], [6], however the RethinQ [7], Lesser-Earth [8] and Echo CRT [9] studies demonstrated poor response and potential harm in narrow QRS patients with echocardiographic MD receiving CRT. The mechanisms underpinning MD in narrow QRS patients and whether such dyssynchrony is amenable to the electrical treatment of CRT remain unclear [10]. Cardiac magnetic resonance (CMR) is not hindered by acoustic windows and offers superior endocardial definition to assess volumes and motion [11]. CMR is the imaging modality of choice for tissue characterization of myocardial scar with late gadolinium enhancement (LGE) [12]. We have shown that CMR derived dyssynchrony measures (standard deviation of time to reach minimal volume for each LV segment; CMR-SDI) are highly reproducible and predicts volumetric response to CRT in patients with prolonged QRS [13]. We hypothesized that MD in heart failure patients with narrow QRS may be due to the presence of myocardial scar rather than as a consequence of electrical dyssynchrony. This would explain why CRT may not be corrective in such cases. To assess this we determined the prevalence of CMR derived measures of MD in systolic heart failure patients with both broad (≥ 130 ms) and narrow QRS duration (< 130 ms). We further assessed whether the presence of LGE-derived left ventricular scar (not amenable to CRT) might explain the presence of mechanical dyssynchrony amongst narrow QRS patients.

## 2 Methods

### 2.1 Inclusion criteria

The local ethics authority approved the study and all patients provided written informed consent; the study complies with the 1975 Declaration of Helsinki. Consecutive patients with NYHA II–IV symptomatic heart failure and an LV ejection fraction (EF) < 35% who were under the care of a dedicated heart failure clinic were included regardless of the QRS duration or morphology. Patients were categorized as broad or narrow QRS based on a QRS duration < 130 ms (NQRS) or ≥ 130 ms (BQRS) on the basis of the RethinQ and Echo-CRT studies [7], [9].

### 2.2 MRI assessment of dyssynchrony and myocardial scar

All subjects underwent a CMR scan using either a 1.5T or a 3T MR-scanner with a 32-element cardiac coil (Achieva, Philips Healthcare, Best, Netherlands). Steady-state free precession (SSFP) imaging was performed to generate a short-axis stack of the entire LV and 2-, 3- and 4-chamber long-axis views of the LV.

### 2.3 Scar assessment

Delayed contrast-enhanced scar imaging was performed 15–20 min following the administration of 0.1–0.2 mmol/kg gadopentetate dimeglumine (Magnevist®, Bayer Healthcare, Dublin, Ireland) using conventional inversion recovery techniques. An ischemic etiology was defined as presence of sub-endocardial scar in two or greater segments. Scar volume was calculated using CMR^42^ (Circle Cardiovascular Imaging Inc., Calgary, Canada) from the CMR scar imaging short axis stack by segmenting the endocardial and epicardial borders and applying a user defined high signal filter in order to highlight scarred regions; this was calculated as percentage of total myocardial mass.

### 2.4 Dyssynchrony assessment

The regional volume change within the LV cavity over the cardiac cycle for 16 segments (defined using the American Heart Association model of the LV) was determined using TomTec 4D LV-Analysis software (TomTec Imaging systems; Unterschleissheim, Germany). The software performs semi-automatic segmentation and propagation of the LV endocardial border from the SA stack and three long axis SSFP cine images [13], [14]. The CMR-SDI was calculated for regional cavity volume change and defined as the standard deviation (SD) of the regional times to peak volume change for the 16 segments. The CMR-SDI was expressed as a percentage of the cardiac cycle to allow for heart rate variation. An SDI of ≥ 9.75% was used as a definition of presence of mechanical dyssynchrony [13].

### 2.5 Statistics

Statistical analysis was performed on PASW Statistics 21 (SPSS Inc., Chicago, IL, USA). The Shapiro-Wilk test was used to ensure variables were normally distributed. Continuous variables were expressed as mean ± SD. Group comparisons were performed using an independent-samples *t*-test for normally distributed data, and the Mann-Whitney *U* test if deemed non-parametric. Nominal variables were expressed as absolute count and percentages and compared with a Chi-squared test or a Fisher's exact test dependent on number. Relationships were assessed using the Pearson correlation coefficient. Values of p < 0.05 were considered statistically significant.

## 3 Results

### 3.1 Baseline characteristics (see Table 1)

A total of 130 patients underwent CMR assessment of mechanical dyssynchrony. Mean age was 64.7 ± 13.8 and 106 (81%) were men. Sixty patients (46%) had an ischemic cardiomyopathy; late gadolinium enhancement imaging was precluded in 6 patients due to renal function. Mean CMR derived EF was 26.8 ± 8.6% and mean QRS duration was 137 ± 30 ms. A left bundle branch block morphology (LBBB) was present in 75 (58%) patients, right bundle branch block (RBBB) in 2 (2%), non-specific intraventricular conduction delay (NIVCD) in 5 (4%) and 48 (36%) had no conduction delay. When dichotomizing the cohort by QRS duration 71 patients had a QRS duration ≥ 130 ms (BQRS group) and 59 had a QRS duration < 130 ms (NQRS group). Patients with NQRS were younger (61.7 ± 14.7 yrs. vs. 67.2 ± 12.5 yrs., p = 0.03). The mean QRS duration in the NQRS group was 109 ± 11 ms and in the BQRS group 160 ± 20 ms (p < 0.01). Six of 59 patients (10%) of the NQRS group fulfilled (non-strict) criteria for LBBB compared to 69/71 (97%) in the BQRS group (p < 0.01) (Table 1).

**Table 1:** Baseline characteristics, scar volume and CMR-SDI for all patients. Values are mean ± SD or n (%). NI-Non-ischemic, I-Ischemic, BQRS – Broad QRS, NQRS – Narrow QRS.

		All (n = 130)	QRS < 130 (NQRS) (n = 59)	QRS ≥ 130 (BQRS) (n = 71)	
Age		64.7 ± 13.8	61.7 ± 14.7	67.2 ± 12.5	p = 0.03
Sex	M	106 (81)	46 (78)	60 (84)	χ^2^ = 0.92 p = 0.34
	F	24 (19)	13 (22)	11 (16)	
Ejection fraction (%)		26.8 ± 8.6	28.2 ± 9.1	25.6 ± 8.0	p = 0.09
QRSd (ms)		137 ± 30	109 ± 11	160 ± 20	p < 0.01
QRS morphology	LBBB	75 (58)	6 (10)	69 (97)	p < 0.01
	RBBB	2 (2)	0 (0)	2 (3)	
	NIVCD	5 (4)	5 (8)	0(0)	
Etiology	NI	70 (54)	29 (49)	41 (58)	χ^2^ = 0.96 p = 0.33
	I	60 (46)	30 (51)	30 (42)	

The mean scar volume for the entire cohort was 11.0 ± 5.6%.

### 3.2 Mechanical dyssynchrony

**Fig. 1:**
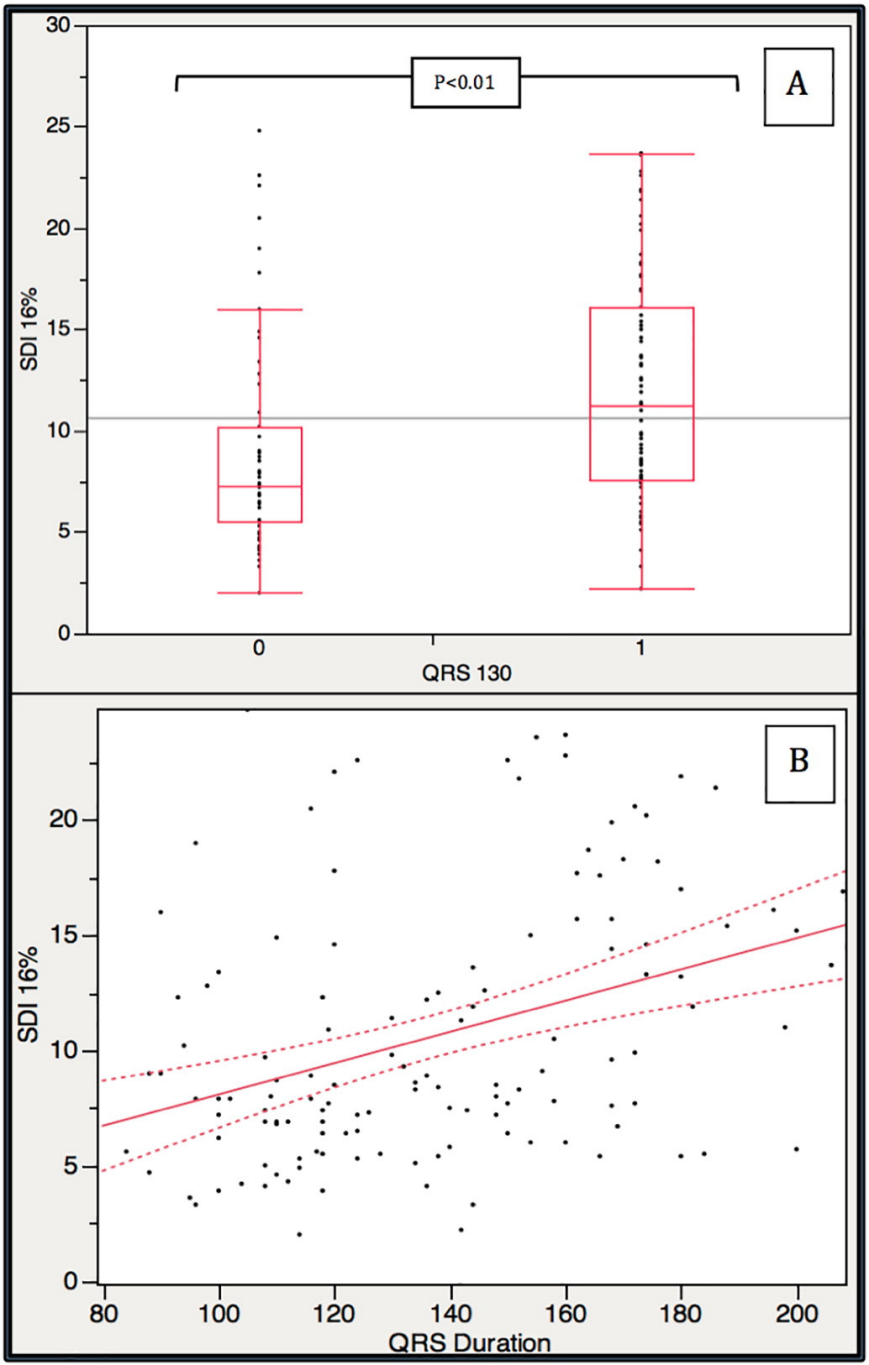
A) Box plot of CMR-SDI (SDI 16%) for patients with a QRS < 130 ms and QRS ≥ 130 ms. Grey horizontal line represents mean for entire cohort. B) Scatter diagram of QRS duration (ms) and Systolic CMR-SDI (SDI 16%). with regression line of best fit and 95% confidence intervals (dashed lines); R = 0.38, p < 0.01.

The mean CMR-SDI was 10.6 ± 5.6% with significant difference between those with NQRS and BQRS (8.9 ± 5.2% vs. 12.1 ± 5.7%, p < 0.01, Fig. 1). On the basis of our pre-specified cut off of SDI ≥ 9.75% MD was present in 25% of NQRS patients and 56% of broad QRS patients. (χ^2^ = 12.62, p < 0.01). There was a modest correlation between QRS duration and CMR-SDI (R = 0.38, p < 0.01) (Fig. 1).

### 3.3 Etiology and scar volume

**Fig. 2:**
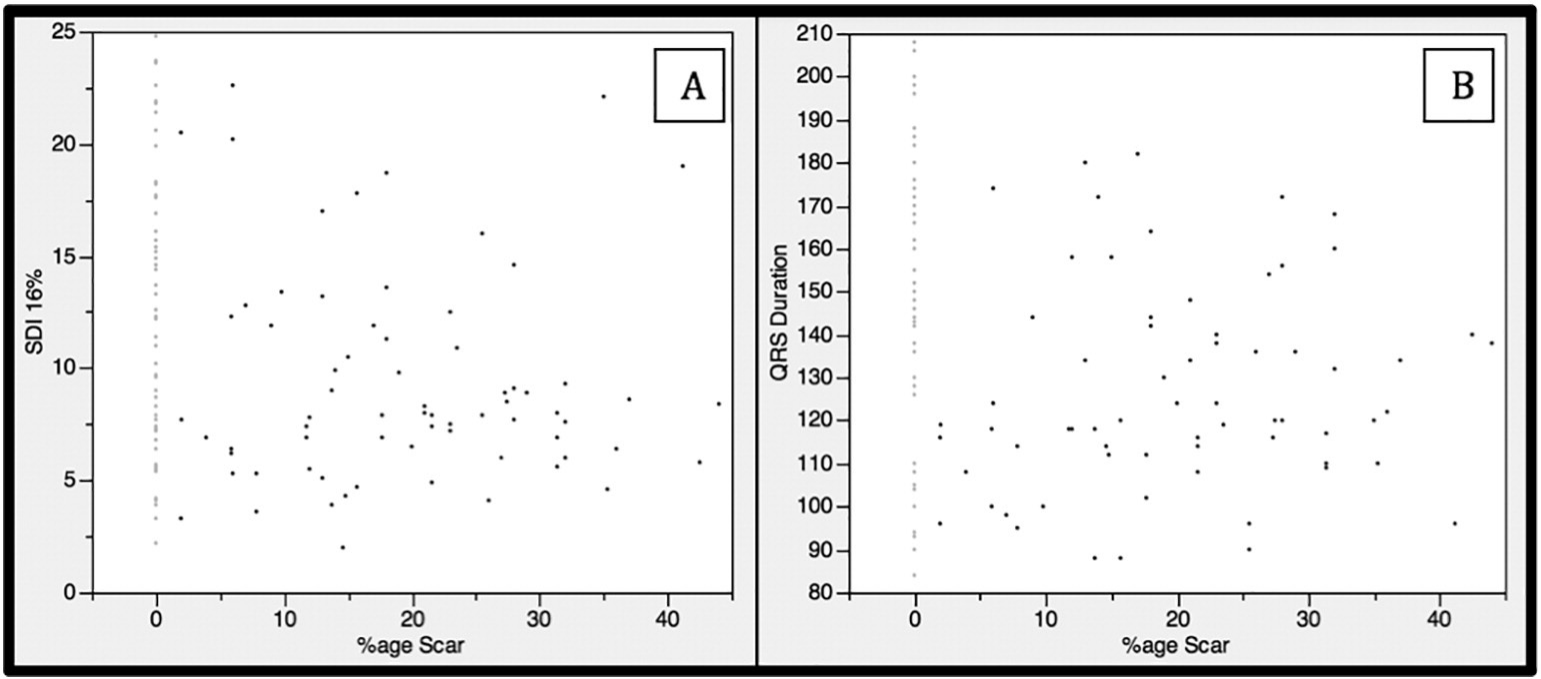
Scatter diagrams of left ventricular scar volume (%age scar) plotted against CMR-SDI (SDI 16%) in A and QRS duration in B. Patients with no scar are represented with grey datapoints.

Ejection fraction (EF) was not significantly different between ischemic and non-ischemic patients. Ischemic patients had narrower QRS durations (131 ± 26 vs. 142 ± 33 ms, p = 0.04) and a lower CMR-SDI (9.4 ± 4.4 vs. 11.6 ± 6.4%, p = 0.03); in keeping with this, patients with CMR defined scar had shorter QRS durations (127 ± 24 vs. 148 ± 34 ms, p < 0.01) and a lower SDI (9.4 ± 4.8 vs. 12.1 ± 6.3%, p < 0.01) without different EFs. There was a significant negative correlation between scar burden and both QRS duration and SDI (R = − 0.23, p = 0.01 & R = − 0.21, p = 0.02 respectively) (Fig. 2).

### 3.4 Relationship between dyssynchrony and scar

**Fig. 3:**
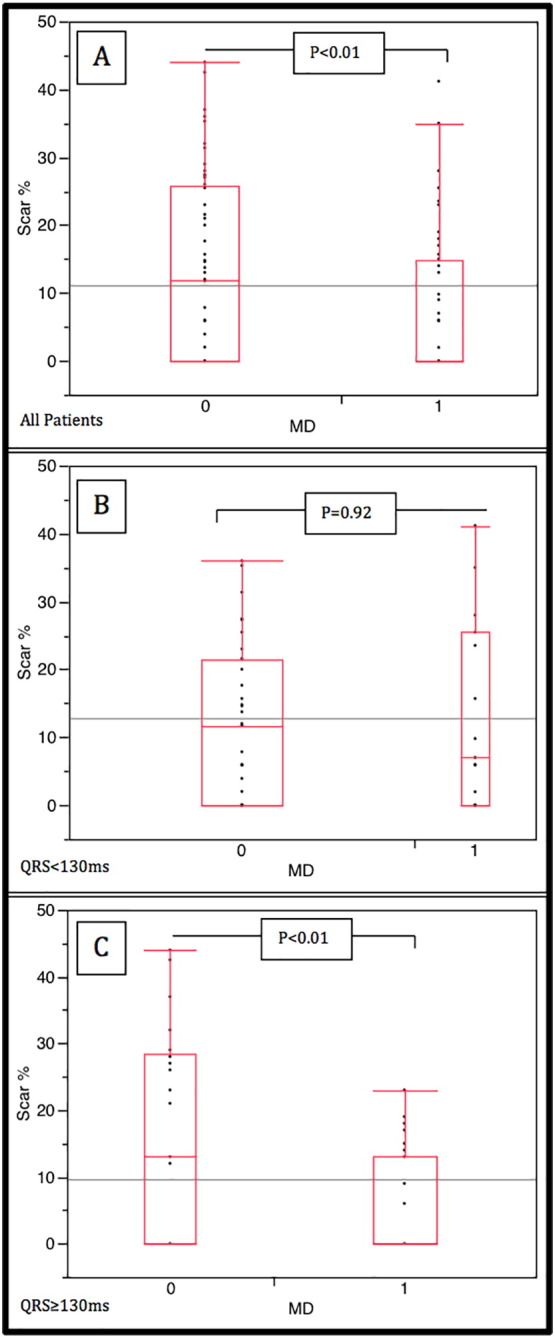
Box plots of left ventricular scar volume (Scar %) for those patients with and without mechanical dyssynchrony (MD). Plot A is all patients, B is narrow QRS patients (QRS < 130 ms), and C is Broad QRS patients (QRS ≥ 130 ms).

Scar volume was compared in patients on the basis of presence or absence of MD. In the whole cohort (incorporating NQRS and BQRS), those patients with mechanical dyssynchrony had a significantly lower scar volume (7.4 ± 10.5% vs 13.7 ± 13.3% vs. p < 0.01). This was the case in the BQRS group with a significantly lower scar burden in patients with MD (5.0 ± 7.7% vs 15.4 ± 15.6%, p < 0.01). Notably in the NQRS group this difference was absent with an equal scar burden in patients with MD 13.3 ± 13.9% and without MD 12.5 ± 11%, p = 0.92 (Fig. 3). These analyses were repeated for non-ischemic and ischemic patients; in non-ischemic patients there was less scar in the BQRS group (1.4 ± 6.8 vs. 3.5 ± 5.9%, p = 0.01) but no difference in amount of scar when analysed for those with and without MD in each group. In ischemic patients there was no difference in scar volume between NQRS and BQRS (21.3 ± 9.8 vs. 22.9 ± 8.4%, p = 0.52), however there was less scar in the BQRS MD patients than those without MD (16.8 ± 3.1 vs. 27.0 ± 8.4%, p < 0.01), this was not the case in the NQRS patients (24.8 ± 12.4 vs. 20.4 ± 9.0%, p = 0.37) (see Supplementary Table 1). Analysis of all patients with MD showed more scar in the NQRS group than in the BQRS group. (13.3 ± 13.9% vs. 5.0 ± 7.7%, p < 0.01).

## 4 Discussion

Our study represents one of the largest experiences of CMR derived indices of MD in heart failure patients.

The main findings of the current study are that:

1. CMR derived MD was present in 25% of systolic heart failure patients with a narrow QRS (< 130 ms).
2. Patients with a broad QRS duration (> 130 ms) and MD had significantly lower scar burden than those without MD.
3. Narrow QRS patients with MD had an equal scar burden compared to those without MD.
4. Broad QRS patients with MD had a significantly lower scar burden than narrow QRS patients with MD.

### 4.1 Prevalence of mechanical dyssynchrony

This study demonstrates that even amongst heart failure patients with a narrow QRS (< 130 ms) there is a significant amount of CMR derived mechanical dyssynchrony occurring in 25% of patients. MD in a quarter of patients with narrow QRS represents approximately 15% of all heart failure patients [1]. This rate of MD is lower than seen in previous echocardiographic based studies [4], [15], [16]. We believe this CMR technique to be more representative of true motion, due to better endocardial tracking, and may therefore CMR derived MD may be more reproducible than echocardiographic measures and more representative of the true incidence of MD in patients with narrow QRS [13].

### 4.2 Scar and mechanical dyssynchrony

The presence of scar is a significant confounder in the association between QRS duration and dyssynchrony. In the whole cohort, patients with a narrow QRS had more scar than those with a broad QRS. We found a significant negative correlation between scar volume and MRI-SDI driven by those patients without scar having a greater MRI-SDI. 97% of patients with broad QRS duration in our study had left bundle branch block (LBBB) whereas only 10% of the narrow QRS group had LBBB.

Those patient with QRS < 130 ms could have non-specific conduction delay secondary to fractionated QRS complexes due to scar supported by the distribution of QRS morphologies between groups as well as the signal for greater scar volumes amongst patients with shorter QRS durations.

For patients with narrow QRS and an ischemic etiology it has previously been supposed that the presence of mechanical dyssynchrony is a consequence of areas of scar leading to regional variability in contractility [15], [17].

### 4.3 Mechanisms of dyssynchrony in narrow and broad QRS and potential importance to CRT response

Our data would be in keeping with the hypothesis that in patients with broad QRS, which is predominantly LBBB, that the mechanical dyssynchrony seen in this patient group is secondary to the electrical dyssynchrony due to the primary electrical disturbance; i.e. there is coupling of excitation and contraction. As the MD is due to the electrical abnormality then this would be amenable to correction with CRT. Our findings are in support of this as CRT is an efficacious treatment in such patients with LBBB. Conversely in the narrow QRS group our hypothesis that mechanical dyssynchrony is caused by myocardial scar is supported by our findings. Despite the prevalence of MD in a quarter of the NQRS patients we studied this was associated with a large scar burden. Left ventricular scar has an adverse effect on CRT response in terms of acute hemodynamic measures [18], chronic remodeling [19], and clinical improvement or mortality [20], [21]. CRT outcomes are adversely affected by total scar volume and the location of scar at the site of the LV lead [12], [22]. We have demonstrated that narrow QRS patients can have mechanical dyssynchrony, however these patients also have left ventricular scar diminishing their ability to respond. The presence of dyssynchrony in the narrow QRS group is likely to be related to scar rather than electrical dyssynchrony and is therefore a substrate not amenable to CRT as there is no correctable electrical delay in these patients, which remains the primary target for CRT treatment. Our findings of MD in narrow QRS patients being caused by scar rather than electrical delay may also provide an insight into the potential for harm from CRT in this patient group as pacing in or adjacent to areas of scar have the potential result in adverse electrophysiological changes which may predispose to reentrant ventricular arrhythmias [23].

Other potential mechanisms to explain the MD seen in our narrow QRS patients include regional variability in multiple cell and molecular pathways including calcium handling proteins, cardiotrophin-1, connexin 43, stress-response kinases and tissue necrosis factor expression [24], [25], [26]. CMR derived T1 mapping gives an insight into the amount of myocardial free water in both the extra cellular volume and the cells and therefore elevated levels represent edema or protein deposition seen in preclinical fibrotic changes [27]. Further investigation with this new technique may indicate whether the pathways involved are likely to be those that modify myocardial architecture or those involved in contractility modulation (i.e. calcium handling proteins as opposed to stress-response kinases).

### 4.4 Limitations

We have described one of the largest MRI studies of dyssynchrony in heart failure patients with the particular interest in assessing the presence and etiology of MD amongst narrow QRS patients. This said the number of narrow QRS patients is relatively small and may not truly reflect the whole population, although this is one of the largest cohorts published in this area. We have not progressed to assess the impact of CMR-SDI defined mechanical dyssynchrony amongst narrow QRS patients on potential response to CRT implantation, however following the publication of Echo-CRT we should be cautious in these studies until we know more about potential treatable mechanisms for mechanical dyssynchrony. One important consideration with respect to the prevalence of mechanical dyssynchrony is that by nature of the fact that the regional volume changes are reduced in systolic heart failure, the measurement of peak volume change is far more difficult because of these lower amplitude permutations. One criticism of the use of CMR as opposed to 2-dimensional echocardiography for SDI is that the temporal resolution is lower which may amplify this difficulty. Although this argument is technically sound, our group has previously shown that in predicting CRT response this CMR-SDI technique outperforms echocardiographic markers of dyssynchrony [13], therefore we feel the benefits it brings in spatial resolution and image clarity more than compensates for this issue. The majority of the BQRS patients were LBBB patients, meaning that right bundle branch block and nonspecific intraventricular conduction delay patients may be under-represented; this may introduce some bias into the dataset. Further work is needed to consider whether other mechanisms such as cellular contractility modulation or electrical remodeling are at play, and ultimately whether these mechanisms are amenable to treatment with CRT which could benefit this significant number of heart failure patients.

### 4.5 Conclusions

A quarter of patients with systolic heart failure and a narrow QRS (< 130 ms) have CMR derived mechanical dyssynchrony. Our findings suggest that MD in this group is secondary to myocardial scar rather than electrical dyssynchrony and therefore not amenable to correction by CRT. Our findings may give insight into the reason for non-response and potential harm from CRT in this patient group.

The following is the supplementary data related to this article.

Supplementary Table 1 Scar volumes amongst all patients, those with and without mechanical dyssynchrony (MD), those with narrow and broad QRS durations, ischemic cardiomyopathy (ICM) and non-ischemic cardiomyopathy (NICM) patients. Values are mean % scar volume ± standard deviation. Supplementary Table 1

## Conflict of interest

The authors declare that they have no conflict of interest.

## Funding sources

This work was supported by the EU FP7 for research, technological development and demonstration under the grant agreement VP2HF [grant number 611823].

The research was supported by the National Institute for Health Research (NIHR) Biomedical Research Centre based at Guy's and St Thomas' NHS Foundation Trust and King's College London. The views expressed are those of the authors and not necessarily those of the NHS, the NIHR or the Department of Health. This work was additionally supported by the Wellcome/EPSRC Centre for Medical Engineering at King’s College London [WT 203148/Z/16/Z].
