# Supplementary material for: The interaction of QRS duration with cardiac magnetic resonance derived scar and mechanical dyssynchrony in systolic heart failure: Implications for cardiac resynchronization therapy
Source: Int J Cardiol Heart Vasc. 2017 Dec 13;18:81–5. doi: 10.1016/j.ijcha.2017.11.005 (PMC5941225; doi:10.1016/j.ijcha.2017.11.005)
Supplement: Supplementary Table 1 — Scar volumes amongst all patients, those with and without mechanical dyssynchrony (MD), those with narrow and broad QRS durations, ischemic cardiomyopathy (ICM) and non-ischemic cardiomyopathy (NICM) patients. Values are mean % scar volume ± standard deviation. [file mmc1.docx]

Supplementary Table

|  | All | | | QRS <130ms | | | QRS ≥130ms | | |
| --- | --- | --- | --- | --- | --- | --- | --- | --- | --- |
| All | 11.0±12.5 | | | 12.7±12.1 | | | 9.6±12.8 | | P=0.03 |
|  | MD | 7.4±10.5 | P<0.01 | MD | 13.3±13.9 | P=0.92 | MD | 4.95±7.7 | P<0.01 |
|  | No MD | 13.7±13.3 |  | No MD | 12.5±11.5 |  | No MD | 15.4±15.6 |  |
| NICM | 2.3±6.5 | | | 3.5±5.9 | | | 1.4±6.8 | | P=0.01 |
|  | MD | 1.3±3.3 | P=0.35 | MD | 3.7±5.5 | P=0.63 | MD | 0.6±2.0 | P=0.98 |
|  | No MD | 3.3±8.6 |  | No MD | 3.5±6.2 |  | No MD | 3.0±11.4 |  |
| ICM | 22.0±9.1 | | | 21.3±9.8 | | | 22.9±8.4 | | P=0.52 |
|  | MD | 19.9±8.8 | P=0.25 | MD | 24.8±12.4 | P=0.37 | MD | 16.8±3.1 | P<0.01 |
|  | No MD | 23.0±9.2 |  | No MD | 20.4±9.0 |  | No MD | 27.0±8.4 |  |

Scar volumes amongst all patients, those with and without mechanical dyssynchrony (MD), those with narrow and broad QRS durations, ischemic cardiomyopathy (ICM) and non-ischemic cardiomyopathy (NICM) patients. Values are mean % scar volume ± standard deviation.
